# Sex differences in metabolic homeostasis, diabetes, and obesity

**DOI:** 10.1186/s13293-015-0033-y

**Published:** 2015-09-03

**Authors:** Franck Mauvais-Jarvis

**Affiliations:** Section of Endocrinology & Metabolism, Department of Medicine, Tulane University Health Sciences Center, 1430 Tulane Avenue, New Orleans, LA 70112 USA

## Abstract

There are fundamental aspects of the control of metabolic homeostasis that are regulated differently in males and females. This sex asymmetry represents an evolutionary paradigm for females to resist the loss of energy stores. This perspective discusses the most fundamental sex differences in metabolic homeostasis, diabetes, and obesity. Together, the role of genetic sex, the programming effect of testosterone in the prenatal period in males, and the activational role of sex hormones at puberty produce two different biological systems in males and females that need to be studied separately. These sex-specific differences in energy homeostasis and metabolic dysfunction represent an untested source of factors that can be harnessed to develop relevant sex-based therapeutic avenues for diabetes, metabolic syndrome, and obesity.

## Review

### Introduction

“This is a man’s, man’s, man’s world but it would be nothing, nothing without a woman or a girl” is sung by James Brown in 1966. Regarding biomedical research, however, it was not until 1993 that the National Institutes of Health (NIH) Revitalization Act mandated the inclusion of women in clinical trials, so that the outcome of all NIH-funded clinical research would generate data that is relevant to individuals of both sexes. The goal was simple: Since a primary aim of research is to provide scientific evidence to improve health, it was imperative to determine whether the therapy being studied differentially affects men and women. Still, most preclinical researchers avoid studying female rodents due to the added complexity of research plans [1], without regard for the consequences of generating data that is mostly relevant to only half of the population. NIH Director Francis Collins and Associate Director for Research on Women’s Health Janine Clayton finally asked scientists to consider sex in preclinical research, to ensure that women get the same benefit of medical research as men [2]. The NIH recently released a notice on new rules to promote the study of animals and cells from both sexes to prevent the overreliance on male animals in preclinical studies [3].

With regard to preventing metabolic disease, there is an urgent need to study both sexes. There are fundamental aspects of metabolic homeostasis that are regulated differently in males and females and likely influence both the development of diabetes and obesity and the response to pharmacological intervention. This perspective reviews the most fundamental sex differences in metabolic homeostasis, diabetes, and obesity, highlights physiological and genetic mechanisms for these sex differences, and proposes that they represent foundations for sex-specific medicine.

### Sex asymmetry in energy metabolism and the gametes

**Fig. 1:**
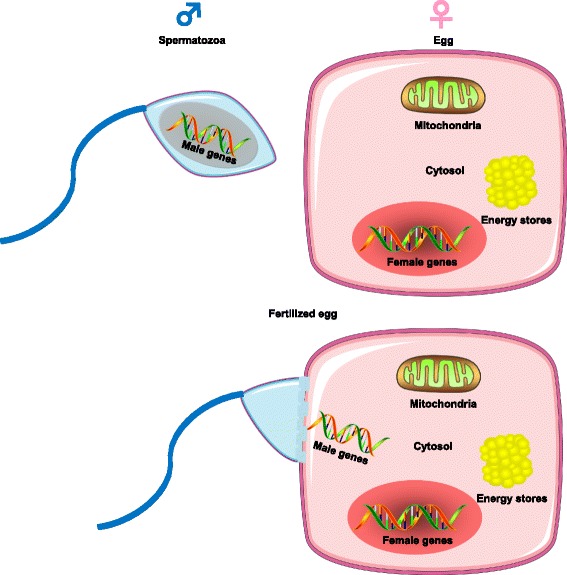
Sex dimorphism in energy metabolism at the level of the gametes. The male spermatozoa (*left*) share only their genes. The female, by way of the fertilized egg (*right*), provides not only her genes but also the energy stores and the nutrients contained in the cytosol of the egg and the mitochondria

Perhaps, the most illuminating sex difference in energy metabolism lies at the level of the gametes themselves, the sperm and the egg [4] (Fig. 1). Males produce sperm that are small, numerous, and highly mobile, but disposable. In contrast, the female produces a small number of large and immobile eggs. While the male shares only his genes during conception, the female, by way of the fertilized egg, provides not only the genes but also the source of energy and the nutrients contained in the cytosol of the egg for the embryo to develop and thrive. Further, the sex dimorphism goes deeper with the uniparental inheritance of the ultimate cellular energy-producing organelle, the mitochondria [5]. The female transmits this critical organelle; the male does not. Thus, from the beginning of reproduction, a major sex asymmetry is present, the fact that females’ gametes transmit all their resources, i.e., their energy stores, their cytosol, and their mitochondria (Fig. 1). It is also noteworthy that female mammals bear the costly burden of gestation and lactation and resist the loss of body energy stores during prolonged periods of food scarcity so that the offspring is not affected. In contrast, in male mammals, energy storage is less an evolutionary strategy. They must mobilize energy stores immediately for short-term and intense muscle activity related to hunting and protection needs. This is also observed at the level of the gametes since spermatozoa are highly mobile cells with a dense mitochondrial network that must provide ATP promptly to its tail to promote energy for sperm mobility [6].

What is the basis for this asymmetry in energy metabolism at the level of the gametes? Sex differences in the gametes do not contribute to sex differences in post-fertilization metabolic phenotypes. However, the sex differences in metabolic homeostasis in somatic tissues, which have evolved to serve female and male differently as we will discuss below, might also have had an impact on germ cells to contribute to the sex differences in gametes. Thus, one may speculate that this sex asymmetry in energy metabolism at the gametes level is a reflection of the adult sexually differentiated metabolic phenotype that is the topic of this review.

### Females have evolved specific ways to favor adipose tissue storage

The first observable consequence of this evolutionary paradigm—that females resist the loss of body energy stores while males mobilize energy stores promptly—is the distribution of the main energy storage organ, the white adipose tissue. Over 70 years ago, Prof. Jean Vague, a French physician from Marseilles, made seminal observations on sex differences in adipose tissue distribution and noted that “A woman normally has twice a man’s fat mass… Though she is as often obese as a man or fatter, she dies less often from the metabolic complications of obesity” [7]. These initial observations led to establish the well-known propensity of women to store adipose tissue in subcutaneous areas compared to the preferential visceral fat deposition in men [8]. These differences can be directly related to the sex-specific role of fat, since subcutaneous adipocytes are more adapted to long-term storage while visceral adipocytes are more metabolically active and sensitive to lipolysis. These sex differences in adipose storage have been recently and extensively reviewed by Susan Fried and Debbie Clegg [9, 10]. The sex difference in adipose storage is not limited to white adipose depots. In animal models, females have more active brown adipose tissue than males [11]. Women also seem to have more brown adipose depots. In a study using (18)F-fluorodeoxyglucose positron-emission tomographic and computed tomographic (PET-CT) scans to identify cervical and thoracic brown adipose tissue, Cypess et al. found that not only were women twice as likely as men to have brown adipose tissue signal, they also had a greater mass of brown adipose tissue with higher metabolic activity [12]. In fact, there is a higher relative contribution of fat mass to resting metabolic rate in women that is in part explained by an increased number of brown adipocytes [13].

The adipose sex dimorphism also extends to the function of adipocytes. The two major adipokines, leptin and adiponectin, are characterized by higher circulating levels in women than men [14, 15]. While some of these differences are related to the action of sex hormones, in the case of leptin, the increase in females is apparent before puberty and remains after menopause [16, 17]. This suggests that factors other than post-pubertal sex hormones are involved. Some of these factors, like genetic sex or prenatal programming by the testicular testosterone surge, will be discussed below.

### Sex differences in energy partitioning and balance

**Fig. 2:**
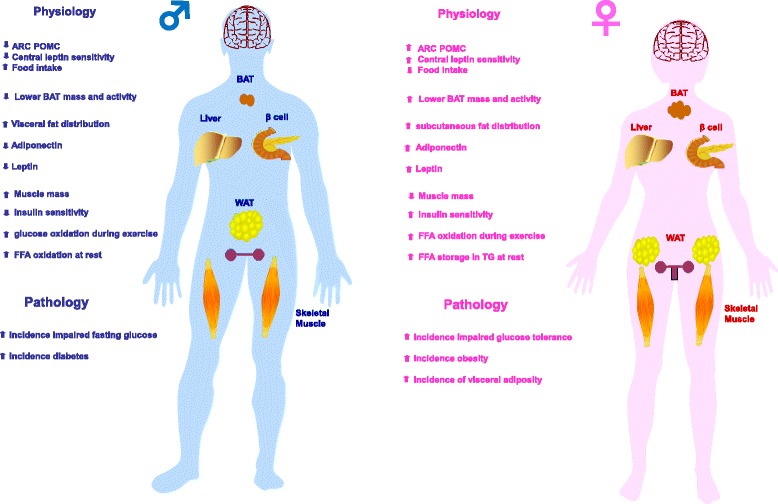
Summary of sex dimorphisms in metabolic homeostasis, diabetes, and obesity. The *left panel* represents the males and the *right panel* the females

Perhaps as a consequence of these evolutionary paradigms, a fundamental sex difference exists in the utilization of carbohydrates and lipids as fuel sources (Fig. 2). At rest and during the post-absorptive state, women tend to incorporate free fatty acids (FFA) into triglyceride (TG) which helps store fat. In contrast, in these same resting conditions, men will oxidize circulating FFA [18]. However, when energy requirements increase during exercise, women oxidize a greater proportion of lipids relative to carbohydrates [19–21]. In contrast, during exercise, men will preferentially use carbohydrates as a fuel source. Women also exhibit a greater reliance on lipids as a fuel source at high altitudes where oxygen is low [22]. In postmenopausal women [23] and ovariectomized female rodents [24], both of whom exhibit estrogen deficiency, lipid oxidation decreases, indicating that estrogen plays a role in this difference. This sex dimorphism in substrate partitioning is dramatically exemplified in mice lacking the peroxisome proliferator-activated receptor α (PPARα^−/−^), the master regulator of fatty acid oxidation. In male PPARα^−/−^mice, pharmacologic inhibition of mitochondrial fatty acid oxidation caused massive lipid accumulation in the liver. As a result of inhibited energy production via fatty acid oxidation, there is excessive glucose oxidation leading to death by hypoglycemia [25]. However, this phenotype was observed in only 25 % of females, suggesting that females can still utilize fatty acids as an energy source. Further, the metabolic phenotype of males was prevented by estradiol treatment. Thus, the female hormone estrogen seems to be a powerful evolutionary mechanism by which, in specific circumstances, females can rely on lipid oxidation as an energy source. This energy partitioning strategy would provide females with a greater capacity to spare protein mass during period of food scarcity, a clear survival advantage [26]. Most importantly, during pregnancy, increased lipid oxidation would also spare circulating glucose away from muscle oxidation in order to preserve it for the fetal brain.

Another fundamental sex difference in energy balance relates to energy intake. In mammals, males consume more food than females, which is considered a masculinized behavior [27]. Although it is assumed that the increased energy intake of males supports their higher muscle mass, the evolutionary survival strategy of male mammals following food deprivation is still to increase fat stores by increasing energy intake [28]. Conversely, females reduce loss of fat stores by decreasing energy expenditure [28]. This latter mechanism is consistent with females’ ability to resist the loss of energy stores during periods of food scarcity. The consequence of these survival strategies is that starvation has a greater negative impact on males. When animals are subjected to complete starvation, females have a greater ratio of lipid to protein loss and are more likely to survive [26, 29]. Anecdotally, the majority of deaths by undernutrition during WWII in Europe were men [30]. Interestingly, this sex difference in the ability to resist the loss of energy stores could result from a sex dimorphism of the hypothalamic melanocortin system, at least in mice. Indeed, compared to females, male mice exhibit a decreased density of anorexigenic neuropeptide pro-opiomelanocortin (POMC) neuronal fibers in the hypothalamic arcuate nucleus, and decreased POMC gene and protein expression, which is associated with an increased energy intake [31]. Accordingly, female rats are more sensitive to the anorectic action of centrally injected leptin than males [32]. Male rats, however, are more sensitive to the anorectic action of insulin injected in the brain [32], an observation that translates to humans. Indeed, intranasal insulin—which reaches the brain directly along olfactory perivascular channels—reduces body fat in men but not in women [33]. We will discuss below how some sex dimorphisms in hypothalamic structure and function can be set by the organizational effect of the prenatal testicular testosterone surge. Figure 2 summarizes sex differences in energy balance.

### Females favor glucose homeostasis

**Fig. 3:**
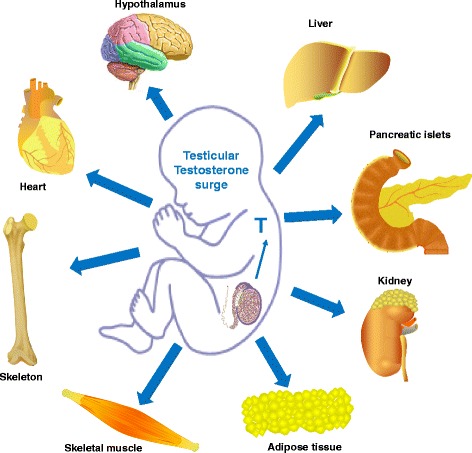
Proposed mechanism for sexual differentiation of metabolism. Perinatal exposure to testosterone in female rodents and primates can program multiple tissues involved in metabolic homeostasis. This could lead to a sexual differentiation or masculinization of metabolic homeostasis

Compared to men, healthy women have more adipose mass, more circulating FFA, higher intramyocellular lipid content, and only two thirds the skeletal muscles mass of men, all factors that would be predicted to promote insulin resistance in women. Yet, women are as sensitive to insulin as men (Fig. 3). In fact, women are resistant to FFA-induced insulin resistance [34]. When matched for physical fitness and under hyperinsulinemic, normoglycemic clamp conditions, whole body insulin sensitivity is 41 % greater in women as a result of a 47 % greater glucose uptake by female skeletal muscles [35]. Physical fitness is an important parameter, and women with lower fitness than men have decreased insulin sensitivity [36]. Yet, these same women exhibit comparable glucose disposal to men because of enhanced glucose effectiveness. The maintenance of glucose homeostasis in women is at least partially due to the beneficial effect of a tight physiological window of estrogen concentrations between puberty and menopause. Indeed, after menopause, when serum estrogen concentrations drop, women become insulin resistant. Similarly, when circulating estrogen concentration rises to supraphysiological levels [24], or when powerful synthetic estrogens like oral contraceptives are used [37], insulin resistance develops.

### Males are predisposed to diabetes

The prevalence of pre-diabetic syndromes such as impaired fasting glucose (IFG) and impaired glucose tolerance (IGT) differs by sex, with IFG being more prevalent in men while IGT is more prevalent in women [38–41]. The reason for these differences is unknown. However, one can speculate that the smaller muscle mass or physical fitness in women discussed above [36] would decrease insulin-stimulated glucose disposal and account for the observed higher rate of IGT. There are also sex differences in diabetes pathophysiology and prevalence. Type 1 diabetes is the only common autoimmune disease characterized by a male predominance in populations of European origin (ratio 1:7) (reviewed in [42]). A strong male predominance (75 %) is also observed in ketosis-prone diabetes (also called idiopathic diabetes), a form of type 2 diabetes with a propensity to acute insulin deficiency [43, 44]. In ketosis-prone diabetes, women are protected unless they are in a hypoestrogenic state [43, 45]. Type 2 diabetes prevalence is also marked by a sex dimorphism. A survey of the global diabetic population revealed that the sex difference in the prevalence of diabetes was reversed depending upon the stage of reproductive life [46]; that is, there are more diabetic men before puberty, while there are more diabetic women after menopause. Figure 2 summarizes sex differences in diabetes and pre-diabetes development.

### Females are predisposed to obesity and metabolic syndrome

As discussed above, females tolerate higher levels of body fat due to a lower amount of abdominal fat. However, they are at greater risk of obesity due to their increased propensity to gain fat. In fact, the global prevalence of obesity is higher in women than in men in all continents [47]. Interestingly, over the last decades, the prevalence of abdominal obesity has increased more in women than in men in the USA [48]. Today, the prevalence of visceral obesity associated with metabolic syndrome is two to ten times higher in women in many countries around the world [49–51]. This female predisposition to central adiposity is observed in many races, across all age groups, and in both urban and rural areas [50]. Figure 2 summarizes sex differences in the incidence of obesity and metabolic syndrome.

### Activational role of gonadal sex hormones after puberty

The major contributors of sex dimorphisms in glucose and energy homeostasis are the “activational” effects of estrogens and androgens acting on their receptors after the onset of puberty. These actions of sex hormones on metabolic homeostasis and their implication for diabetes and obesity in males and females have been extensively described in recent reviews and will not be discussed here [24, 52]. However, it is important to stress that non-reproductive organs like the brain and adipose tissue are also sites of androgen and estrogen biosynthesis. In these tissues, androgens convert to estrogens in both males and females. Adipose tissue is also a reservoir of sex steroids that act locally in a paracrine and intracrine manner (reviewed in [53]). The total sex steroid content of adipose tissue has been estimated in some studies to be a hundred times greater than total plasma content [54]. Steroid fatty acid esters are present in adipose tissue of both male and female rats [55]. These testosterone esters are long-lived in the blood even after castration and represent an adipose testosterone reservoir for slow release (although human adipose tissue steroid ester has not yet been demonstrated). Further, tissue metabolism or inactivation of estrogen by the cytosolic enzyme estrogen sulfotransferase (EST) is also an essential factor in controlling cellular estrogenic action and sex differences in adipose biology; [56, 57]. Thus, in human adipose tissue (and in the brain), the local tissue and cellular output of sex hormone action is more complex than would be predicted by simple measurement of serum sex hormone concentrations in males and females. Therefore, the intracrinology of sex hormones in metabolic tissues in adults is an underestimated but critical aspect of sex differences in metabolic function that should be considered (Fig. 3).

### The organizational role of testosterone surge and the masculinization of metabolism

In mammals, the effect of sex hormones is not limited to their “activational” role after puberty. To achieve a male-specific brain, the differentiated testis produces a prenatal (in human and nonhuman primates) or neonatal (in rodents) testosterone surge that is critical to masculinize the organism [58]. The programming or “organizational” role of testosterone in perinatal life is paramount. If the brain is exposed to testosterone either from its own testes in males or from an exogenous source in females, it will be masculinized [59–64]. Extensive evidence links sexually dimorphic aspects of physiology to differences in programming by this early testosterone surge in males that programs the organization of neural circuits permissive to the activation of male behavior at puberty [59–64]. It is believed that perinatal testosterone exposure causes the hypothalamus to change its structure and function, leading to major sex differences in sexual behavior and reproduction. Thus, like the hypothalamic control of reproduction, the hypothalamic control of energy homeostasis could be programmed or defeminized as well as masculinized by the perinatal testicular testosterone surge in males. In fact, evidence suggests that this is the case for the hypothalamic melanocortin system, at least in mice [31, 65, 66]. Indeed, when female mice are neonatally androgenized with testosterone (as males are normally by the testicular testosterone surge), these neonatally androgenized females exhibit a masculinized POMC neuronal architecture in the hypothalamic arcuate nucleus in adults as is observed in littermate adult males [31]. Further, a transient perinatal androgen excess in female rodents, nonhuman primates, and even humans can masculinize their metabolism and reprogram their genetic predisposition to obesity and metabolic syndrome in adulthood without any alteration in adult sex hormone levels [31, 65–71]. This masculinization involves all the sexually dimorphic parameters discussed above such as adipose tissue distribution, adipocyte size, adipocytokine concentrations, insulin sensitivity, energy intake, and even some aspects of sympathetic tone to adipose tissue [31, 65, 66]. Interestingly, such a masculinization also involves an increase in lean mass of skeletal and cardiac muscles, bone, and kidney [41]. Therefore, the perinatal testosterone surge could program the defeminization and masculinization or a sexual differentiation of metabolic homeostasis (Fig. 3). Further studies are needed to elucidate this critical issue.

### Role of sex chromosomes

Finally, there is the influence of the genetic sex. First, there is what is called the direct sex chromosome effect of X and Y genes that produce differences in cellular function of XX females and XY male cells. For example, the Y chromosome is known to accelerate cellular glucose metabolism and growth [72]. In addition, XY embryos show higher metabolic rates than XX embryos and develop faster [73]. Further, recent evidence suggests that the X and Y chromosomes program sex differences in metabolic regulation [74]. Reue and coworkers used a mouse model in which the gonadal sex (*Sry* gene) and the chromosomal sex (complement of sex chromosomes) are dissociated because Sry is deleted from the Y chromosome and inserted as a transgene into an autosome in both XX (XX*Sry*) and XY (XY^−^*Sry*) genotypes. These mice produce offspring that include XX and XY males (mice that have *Sry* and develop testes) and XX and XY^−^ females (mice that lack *Sry* and develop ovaries). This model system is unique in that it permits testing the independent contribution of *Sry*, which is mediated by testosterone secretion, vs. non-*Sry* sex chromosome genes (complement of sex-linked genes), which have been untested in previous metabolic studies. Using this model, Chen et al. showed that male and female XX mice exhibit increased fat depots and increased food intake compared to XY mice. When placed on a high-fat diet, XX mice gained adiposity and developed fatty liver and insulin resistance regardless of their original gonadal sex. Interestingly, XX mice had larger subcutaneous inguinal adipose tissue depots, whereas XY mice had larger gonadal fat pads, suggesting that the number of X chromosomes contributes to sex differences in fat distribution. In another strain of mice, the same authors showed a role for the Y chromosome in metabolism independent of testes and gonadal hormones [75]. Therefore, both X and Y chromosomes affect adiposity. Consistent with this possibility, neonatally androgenized female mice develop many of the features of metabolic syndrome [31, 65, 66]. These features include increased food intake and lean mass, visceral adiposity with enlarged adipocytes, hypoadiponectinemia, decreased osteocalcin activity, insulin resistance, pre-diabetes, and hypertension. In contrast, littermate male mice also exposed neonatally to testosterone develop a mild metabolic phenotype with decreased lean mass and food intake and SC adiposity without cardiometabolic alterations [76]. Therefore, neonatal testosterone exposure has programmed a different phenotype in male and female adults possibly in relation to their sex-specific genetic background. However, in this model, genetic sex is confounded with hormonal sex. Indeed, males had already been masculinized by their own neonatal testosterone surge, and they may have responded differently to injected testosterone.

## Conclusions

**Fig. 4:**
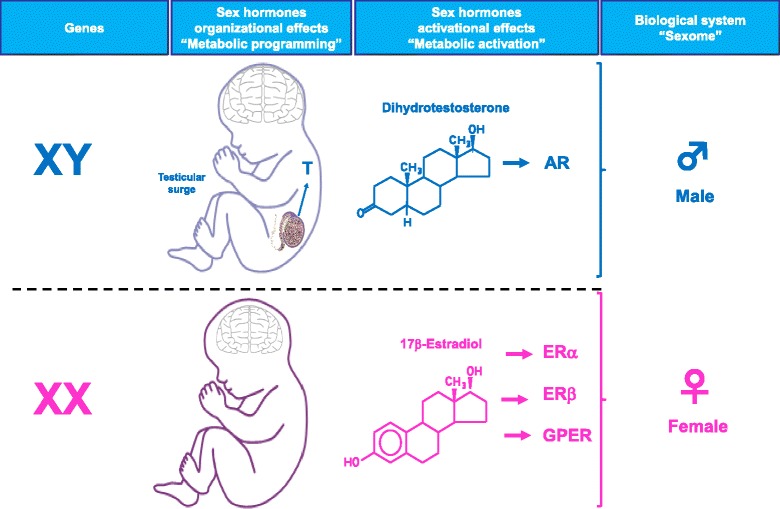
The male and female biological systems. The combined influence of the genetic sex, the organizational effect of the testosterone surge, and the activational role of sex hormones at puberty define two different genetic and biological systems in males and females

Until recently, most clinical research has been carried out under the assumption that the male can fulfill the function of the representative of the human species. Today, basic research using animal models is still performed mostly on males. However, as discussed in this perspective, the role of sex is a fundamental issue in the incidence and evolution of diabetes, obesity, and metabolic syndrome. The fact that many investigators avoid studying females to keep the experimental protocol more simple is in itself admission of sex differences. Together, the role of genetic sex, the organizational role of testosterone in the prenatal period, and the activational role of sex hormones at puberty define two different genetic and biological systems in male and females (Fig. 4). This “sexome,” as coined by Art Arnold and which is the sum of all sex-specific influences on gene networks and cellular systems [77], produces the emerging phenotypical sex differences in body composition and the mechanisms of energy homeostasis. Therefore, we need to study the system biology of each sex globally in order to have an appreciation of the sex-specific aggregate behavior of the genome. Indeed, transcriptome analysis of liver, muscle, and adipose tissues of mice reveals sex differences in expression of over half these genes [78]. Further, a metabolomic profiling of sex-specific differences in serum metabolite revealed major concentration differences between males and females for over three quarters of the metabolites studied [79]. Not surprisingly and as a result, there is a sex-specific response to anti-diabetic drugs in patients with type 2 diabetes. For example, males responded better to sulfonylurea treatment compared with women [80] and responders to rosiglitazone were more likely to be females than males [81].

Since most sex differences related to diabetes and obesity in humans are also found in animal models, both sexes should be studied to elucidate the determinants of these fundamental biological sex differences from genes to hormones. This is a necessary step toward personalized, sex-specific medicine. In fact, there is a clear economic advantage in considering both males and females early in preclinical and translational research. Indeed, the identification of sex-specific differences in metabolic function and metabolic disease incidence and progression would provide an untapped source of factors that can be studied to prevent metabolic dysfunction and inform clinical trials. This knowledge will allow the development of relevant sex-based therapeutic avenues for diabetes, metabolic syndrome and obesity.
